# Bioactivity and Control Efficacy of the Novel Antibiotic Tetramycin against Various Kiwifruit Diseases

**DOI:** 10.3390/antibiotics10030289

**Published:** 2021-03-10

**Authors:** Qiuping Wang, Cheng Zhang, Youhua Long, Xiaomao Wu, Yue Su, Yang Lei, Qiang Ai

**Affiliations:** 1 Department of Food and Medicine, Guizhou Vocational College of Agriculture, Qingzhen 551400, China; qpwang518@aliyun.com (Q.W.); suyue09136@163.com (Y.S.); gznzyylei@126.com (Y.L.); gznzyaqiang@163.com (Q.A.); 2 Research Center for Engineering Technology of Kiwifruit, Institute of Crop Protection, College of Agriculture, Guizhou University, Guiyang 550025, China; chengz76@aliyun.com (C.Z.); wuxm827@126.com (X.W.)

**Keywords:** tetramycin, antimicrobial activity, kiwifruit disease, conventional antibiotics, storage quality

## Abstract

Tetramycin, a novel polyene agriculture antibiotic, has excellent antimicrobial activity against many plant pathogens. In this study, the antimicrobial activities of tetramycin and conventional antibiotics on eight common pathogens and their field control efficacies against four serious diseases in kiwifruit were investigated. The results show that 0.3% tetramycin aqueous solutions (AS) exhibited the superior antibacterial and antifungal activity against *Pseudomonas syringae* pv. *actinidiae*, *Pseudomonas fulva*, *Agrobacterium tumefaciens*, *Botryosphaeria* *dothidea*, *Phomopsis* sp., *Alternaria tenuissima*, *Armillariella mellea* and *Phytophthora cactorum* of kiwifruit pathogens with EC_50_ values of 1.21, 1.24, 0.72, 0.14, 0.09, 0.16, 0.06 and 0.17 mg kg^−1^, respectively. These EC_50_ values of tetramycin were much higher than those of conventional kasugamycin, zhongshengmycin or polyoxin. Meanwhile, 0.3% tetramycin AS possessed the good field control efficacies for canker, soft rot, blossom blight and brown spot disease of kiwifruit with 74.45, 83.55, 84.74 and 89.62%. Moreover, 0.3% tetramycin AS application notably increased fruit resistance substances contents, activated fruit superoxide dismutase and polyphenoloxidase activities, as well as remarkably enhanced fruit growth, improved fruit quality and storability. This study highlights that tetramycin can be used as a preferred alternative to conventional antibiotics in kiwifruit production.

## 1. Introduction

Kiwifruit (*Actinidia*), an emerging, healthy and economical fruit, has been commercially cultivated worldwide on a large scale since the 1970s. The yield and area of kiwifruit cultivation around the world are continuously increasing in the 21st century, and its planting area and annual output reached 381,800 hm^2^ and 5,270,000 tons by 2020 [1,2]. However, as commercial cultivation of kiwifruit expanded, many diseases gradually appeared in kiwifruit orchards and have become increasingly prominent and serious problems [3]. These serious diseases include bacterial canker, soft rot, bacterial blossom blight, brown spot and root rot, etc. For instance, *Pseudomonas syringae* pv. *actinidiae* (*Psa*) is the causal agent of destructive canker disease in kiwifruit, whose field symptoms include leaders and trunks often accompanied by oozing exudates, shoot wilting, reddening of the lenticels, twig dieback, blossom necrosis and leaf spotting [4,5,6]. Soft rot, which mainly caused by *Botryosphaeria dothidea*, *Phomopsis* sp., *Cryptosporiopsis actinidiae*, *Botrytis inereal*, *Cylindrocarpon* sp. and *Phoma exigua*, is a major disease of postharvest kiwifruit [7,8,9,10,11,12]. The occurrence of these diseases seriously affects the quality and yield of kiwifruit, as well as being a frequent cause of major economic losses worldwide.

Recently, China’s kiwifruit industry has developed rapidly, and its planting area has reached over 243,000 hm^2^, with an annual output of nearly 2,500,000 tons. In Guizhou Province of Southwest China, the kiwifruit industry has made great contributions to poverty alleviation and rural revitalization, and its planting area reaches over 40,000 hm^2^. Various diseases including canker (*Psa*), soft rot (*B. dothidea* and *Phomopsis* sp.), blossom blight (*Pseudomonas fulva*), brown spot (*Alternaria tenuissima*), crown gall (*Agrobacterium tumefaciens*) and root rot (*Armillariella mellea* and *Phytophthora cactorum*) are common diseases in kiwifruit planted in Guizhou [12,13,14,15,16]. Although some chemical fungicides have good antibacterial activity against these pathogens, there are increasing concerns about the harmful impacts of chemical fungicide residues on human health and the environment. Moreover, the number of effective control fungicides for these diseases is extremely limited, as for example, only streptomycin or copper are effective in controlling bacterial canker [17]. Additionally, chemical fungicides easily induce pathogen resistance [17,18]. Therefore, there is an urgent need to develop safe and effective control technologies for kiwifruit diseases.

**Figure 1:**
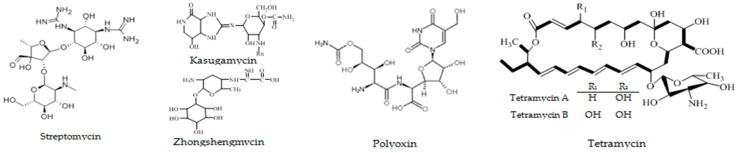
The chemical structures of streptomycin, kasugamycin, zhongshengmycin, polyoxin and tetramycin.

Agricultural antibiotics, as a kind of important biological pesticides, have many prominent advantages including high efficiency, easy decomposition, no residue and no environmental pollution, are thus a green, popular and eco-friendly approach to controlling plant diseases [19,20]. Some conventional antibiotics such as streptomycin, kasugamycin, zhongshengmycin and polyoxin (Figure 1), have been widely used to control various plant diseases [17,19,20,21,22]. However, we found that these conventional antibiotics exhibited low efficacy in controlling many diseases of kiwifruit, and streptomycin application in agricultural production in China has been already limited. Tetramycin, produced by *Streptomyces hygrospinosus* var. *Beijingensis*, is a novel 26-member tetraene macrolide antibiotic containing two active components (tetramycin A and tetramycin B, Figure 1) [23,24]. It exhibits satisfactory inhibitory bioactivity against numerous plant pathogens, such as *Botrytis cinerea*, *Colletotrichum scovillei*, *Pyricularia oryzae*, *Phytophthora capsici* and *Passalora fulva* [25,26,27,28,29,30]. Recently, tetramycin was registered for controlling rice and fruit diseases in China [25], and has gradually become a preferred alternative to conventional antibiotics because of its environmental friendliness and low toxicity [31,32]. Up to date, however, there is little attention paid or documentation available about the application of tetramycin for the control of various kiwifruit diseases.

Accordingly, this study was initiated to evaluate the antimicrobial activity of tetramycin and conventional antibiotics against eight common pathogens of kiwifruit, and to assess the control efficacy of tetramycin and the conventional antibiotics against canker, soft rot, blossom blight and brown spot diseases of kiwifruit under field conditions. Moreover, the effects of tetramycin on the disease resistance, growth and quality of kiwifruit were investigated. The findings should provide a technical basis for the registration and application of tetramycin for the control of various kiwifruit diseases.

## 2. Materials and Methods

### 2.1. Pathogens and Materials

*Psa, B. dothidea*, *Phomopsis* sp., *P. fulva*, *A. tenuissima*, *A. tumefaciens*, *A. mellea* and *P. cactorum* were provided by the Research Center for Engineering Technology of Kiwifruit, Guizhou University (Guiyang, China), and they had highly pathogenicity. 0.3% tetramycin aqueous solutions (AS) was purchased from Liaoning Microke Biological Engineering Co. Ltd. (Liaoning, China). 5.0% polyoxin AS was obtained from Rushan Hanwei Biotechnology Co., Ltd. (Shandong, China). 4.0% kasugamycin wettable powder (WP) was purchased from Huafeng Chemical Co., Ltd. (Qiqihar, China). 3.0% zhongshengmycin WP was obtained from Noposion Agrochemicals Co., Ltd. (Shenzhen, China). Potato dextrose agar (PDA, potato 200 g, dextrose 20 g, agar 15 g, distilled water 1000 mL) and nutrient agar (NA, beef extract 5.0 g, peptone 10.0 g, NaCl 5 g, distilled water 1000 mL) were purchased from Xiya Reagent Co. Ltd. (Chengdu, China).

### 2.2. In Vitro Toxicity Tests

For bacterial pathogens (*Psa*, *P. fulva* and *A. tumefaciens*), the plate colony counting method was used to determine the in vitro toxicity of bactericides. 1 mL tested solution of bactericide and 9 mL NA were emptied into the glass petri dishes (90 mm in diameter) and mixed, 1 mL sterile water was used as control. After solidification, 200 μL bacterial suspension (1000 cfu mL^−1^) was evenly coated on the NA plate containing bactericide with three replicates, and then cultured at 28 °C for 48 h. The colony number of each replicate was observed and counted. The formula for calculating the inhibition rate of bacteria was as Equation (1):

Inhibition rate (%) = 100 × (Colony counts in control dish − Colony counts in treatment dish)/ Colony counts in control dish(1)

EC_50_ (effective concentration of 50% inhibition rate) values were estimated statistically using the SPSS 18.0 software.

For fungal pathogens (*B. dothidea*, *Phomopsis* sp., *A. tenuissima*, *A. mellea* and *P. cactorum*), the mycelial growth rate method was used to determine the in vitro toxicity of fungicides. 9 mL PDA was emptied into the glass Petri dishes. After PDA solidification, 1 mL tested solution of fungicides was evenly coated on the PDA plate, sterile water was used as control. Then, a 5 mm diameter disc of pathogen which cut from the actively growing front of a 7 d old colony was placed in the plate center with the inoculum side down and three replicates. Subsequently, the treated plate cultured at 28 °C till the fungal growth was almost complete in the control plates, the diameters of the fungal growth were measured. The formula for calculating the growth inhibition of fungal hyphae was as Equation (2):

Inhibition rate (%) = 100 × [(Mycelial growth diameter in control dish − Mycelial growth diameter in treatment dish)/(Mycelial growth diameter in control dish − 5)](2)

The calculation of EC_50_ values was the same as above.

### 2.3. Field Experiments

#### 2.3.1. Study Site

Field experiments were conducted in a kiwifruit garden at Xiuwen Country, Guizhou, China (26°79′80.0″ N, 106°56′58.2″ E), where serious infestations of bacterial canker, soft rot, bacterial blossom blight and brown spot of kiwifruit had occurred in previous years. The cultivar was *A. deliciosa* cv. Guichang, with a tree age of 5 years and spacing of 3.0 m × 3.0 m, cultivated on concrete ‘T’ type frames. The proportion of male and female kiwifruit plants was 1:8. The annual rainfall, mean temperature and altitude of the kiwifruit garden was about 1293 mm, 15~16 °C and 1267 m, respectively. The loam soils (0~60 cm in deep) had 25.94 g kg^−1^ of organic matter, 1.38 g kg^−1^ of total nitrogen, 1.65 g kg^−1^ of total phosphorus, 1.08 g kg^−1^ of total potassium, 3.96 mg kg^−1^ of available nitrogen, 4.25 mg kg^−1^ of available phosphorus, 3.15 mg kg^−1^ of available potassium, 31.61 mg kg^−1^ of available iron, 19.17 mg kg^−1^ of available manganese, 50.66 mg kg^−1^ of total zinc, 17.63 cmol kg^−1^ of exchangeable calcium, and 5.72 of pH value.

#### 2.3.2. Field Experiment Design of Kiwifruit Canker

The bactericide control experiment of kiwifruit canker was carried out using the smearing disease spot method. The experimental treatments included 0.3% tetramycin AS 50 times dilution liquid, 3.0% zhongshengmycin WP 50 times dilution liquid and clear water (control), and a total of nine plots were arranged randomly with three replicates (the plot distribution figure see Figure S1 in the Supplementary Materials). Each plot had eight kiwifruit trees, and the interior six trees were used for determination. The bactericide was applied in mid-March for three times with an interval of 7 d. The healing rate of the disease spot was observed and recorded after the three months of application, and the healing rate and control effect were calculated according to Equations (3) and (4):

Healing rate (%) = 100 × Number of healed disease spots after treatment/Total number of disease spots before treatment (3)

Control effect (%) of canker = 100 × (Healing rate of control − Healing rate of treatment)/ (100 − Healing rate of control)(4)

#### 2.3.3. Field Experiment Design of Soft Rot, Blossom Blight and Brown Spot Diseases in Kiwifruit

The fungicide control experiment of soft rot, blossom blight and brown spot diseases in kiwifruit was carried out using the spray method. The experimental treatments included 0.3% tetramycin AS 5000 times dilution liquid, 5.0% polyoxin AS 5000 times dilution liquid and clear water (CK), and a total of nine plots were also arranged randomly with three replicates (the plot distribution figure see Figure S2 in the Supplementary Materials). Similarly, each plot had eight kiwifruit trees, and the interior six trees were used for determination. In our previous report, the pathogens of soft rot have the two infection periods on *A.delicios*. cv. Guichang, one is 20 May to 13 June and other is 2 to 12 August [16]. Moreover, blossom blight often occurs in the flower bud stage (late March to late April), and brown spot often occurs in the fruit growth stage (early June to late August). Thus, about 1.00, 1.50 and 2.00 L of fungicide dilution liquid was sprayed on kiwifruit plants (include bud, leaf and stem) at 19 March, 19 May and 1 August in 2020, respectively. The incidence rate of disease flower bud was observed and recorded at flowering phase (15 April to 15 May), and the incidence rate and control effect of blossom blight in kiwifruit were calculated according to Equations (5) and (6):

Incidence rate of disease flower buds (%) = 100 × Number of disease flower buds/ Total number of flower buds (5)

Control effect (%) of blossom blight = 100 × (Incidence rate of control − Incidence rate of treatment)/ Incidence rate of control(6)

The disease index and control effect of brown spot in kiwifruit were investigated at 15 August according to Equations (7) and (8). The disease grade of 10 leaves of 6 branches in each plot was observed and recorded. The grading standard of the incidence degree: 0 = no incidence; 1 = disease spot area is less than 10% of leaf area; 2 = disease spot area is 10~20% of leaf area; 3 = disease spot area is 20~40% of leaf area; 4 = disease spot area is more than 40% of leaf area; 5 = fallen leaf.

Disease index = 100 × ∑(Disease grade value × Number of leaf within each grade)/ (Total number of leaf × the highest grade) (7)

Control effect of brown spot (%) = 100 × (Disease index of control − Disease index of treatment)/ Disease index of control (8)

Two hundred kiwifruits from each plot were randomly collected and divided into two groups at 1 October in 2020, and stored at 25 ± 1 °C. Fruits of the first group were used to investigate the incidence rate and of control effect soft rot in kiwifruit. Fruits of other group were used for determining the development and quality parameters of fruit. The incidence rate and control effect of soft rot in kiwifruit were investigated according to Equations (9) and (10).

Incidence rate of disease fruits (%) = 100 × Number of disease fruits/Total number of fruits (9)

Control effect (%) of soft rot = 100 × (Incidence rate of control − Incidence rate of treatment)/ Incidence rate of control (10)

Because it is difficult to find the continuous occurrence garden of crown gall and root rot diseases in kiwifruit, thus the field control experiments of tetracycline against crown gall and root rot diseases were not carried out.

### 2.4. Analytical Methods

Total phenolics, total flavonoids, superoxide dismutase (SOD) activity and polyphenoloxidase (PPO) activity of fruits were analyzed according to Zhang et al. [15,16]. The development parameters including longitudinal diameters, transverse diameters, lateral diameters, fruit shape index, single fruit volume and single fruit weight, and the quality parameters including vitamin C, total soluble sugar, soluble solid, dry matter, soluble protein, titratable acidity and fruit firmness were also analyzed as described by Zhang et al. [15,16].

### 2.5. Statistical Analyses

The mean and standard deviation values of triplicate were presented. All analyses were performed using SPSS statistical software package release 18.0 (SPSS Inc., Chicago, IL, USA). The difference significances between group means were treated statistically by one-way analysis of variance (ANOVA). Charts were plotted with Origin 10.0 (OriginLab Inc., Northampton, MA, USA).

## 3. Results

### 3.1. Toxicity Effects of Different Antibiotics against Eight Pathogens of Kiwifruit

The toxicities of tetramycin, polyoxin, kasugamycin or zhongshengmycin against eight pathogens of kiwifruit are shown in Table 1. 0.3% Tetramycin AS exhibited a superior toxicity potential for *Psa*, *P. fulva* and *A. tumefaciens* of bacterial pathogens in kiwifruit with EC_50_ values of 1.21, 1.24 and 0.72 mg kg^−1^, which were 101.91, 99.12 and 721.17 folds, or 14.01, 209.06 and 3905.39 folds higher than 4.0% kasugamycin WP or 3.0% zhongshengmycin WP, respectively. 0.3% tetramycin AS caused the greatest toxicities of mycelium growth for *B. dothidea* and *Phomopsis* sp. of kiwifruit soft rot with EC_50_ values of 0.14 and 0.09 mg kg^−1^, which were 603.36 and 438.67 folds, or 5158.64 and 14515.11 folds higher than 5.0% polyoxin AS or 4.0% kasugamycin WP, respectively. Moreover, 0.3% tetramycin AS also possessed superior toxicities for *A. tenuissima*, *A. mellea* and *P. cactorum* of other fungal pathogens in kiwifruit with EC_50_ values of 0.16, 0.06 and 0.17 mg kg^−1^, which were 91.06, 272.33 and 56.59 folds, or 8513.19, 9306.83 and 4665.71 folds higher than 5.0% polyoxin AS or 4.0% kasugamycin WP, respectively. These results suggest that tetramycin had an extremely superior antimicrobial activity than conventional antibiotics such as kasugamycin, zhongshengmycin and polyoxin.

### 3.2. Field Control Effects of Tetramycin on Canker Disease of Kiwifruit

Table 2 exhibits the field control effects of tetramycin and zhongshengmycin on canker disease of kiwifruit. Prominently, the healing rate of disease spots in kiwifruit by 0.3% tetramycin AS was 72.68%, which was significantly (*p* < 0.01) higher than that of 3.0% zhongshengmycin WP (50.23%) and control (3.95%). Satisfactorily, the application of 0.3% tetramycin AS exhibited a good control capacity for canker disease of kiwifruit with control effect of 74.45%, which was significantly (*p* < 0.01) higher than 45.24% of 3.0% zhongshengmycin WP.

### 3.3. Field Control Effects of Tetramycin on Soft Rot, Blossom Blight and Brown Spot Diseases of Kiwifruit

The field control effects of tetramycin and polyoxin on soft rot, blossom blight and brown spot diseases of kiwifruit are displayed in Table 3. 0.3% Tetramycin AS and 5.0% polyoxin AS significantly (*p* < 0.01) decreased the incidence rates of disease fruit and flower bud in kiwifruit, and significantly (*p* < 0.01) decreased disease index of brown spot diseases in kiwifruit, as well as 0.3% tetramycin AS was more effective than 5.0% polyoxin AS. The control effects of soft rot, blossom blight and brown spot diseases in kiwifruit by 0.3% tetramycin AS were 83.55, 84.74 and 89.62%, which were significantly (*p* < 0.01) higher than 60.83, 34.73 and 55.51% of 5.0% polyoxin AS, respectively. These results indicate that 0.3% tetramycin AS had a superior prevention and control capacity for various diseases in kiwifruit production.

### 3.4. The Effects of Tetramycin on Defense-Related Substances and Enzyme Activity in Kiwifruit

**Figure 2:**
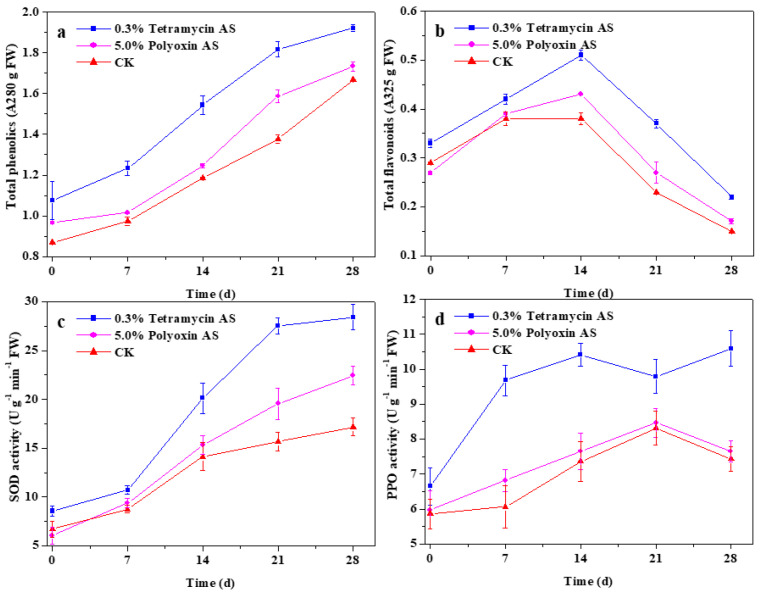
The effects of tetramycin and polyoxin on the changes of total phenolics (**a**) and total flavonoids (**b**), SOD activity (**c**) and PPO activity (**d**) in kiwifruit during storage. Values indicate the mean of three replicates, error bars indicate SD of three replicates.

Figure 2 depicts the effects of tetramycin and polyoxin on the changes of total phenolics and total flavonoids, SOD activity and PPO activity in kiwifruit during storage. Total phenolics content increased gradually during storage, and total phenolics content of 0.3% tetramycin AS-treated fruits was consistently higher than that of 5.0% polyoxin AS-treated and control fruits (Figure 2a). Total flavonoids content in kiwifruit increased gradually over the first 14 d and then decreased on the second 14 d, and total flavonoid content of 0.3% tetramycin AS-treated fruits was also consistently significant (*p* < 0.01) higher than that of 5.0% polyoxin AS-treated and control fruits (Figure 2b). Similarly, SOD and PPO activities of 0.3% tetramycin AS-treated fruits were consistently significant (*p* < 0.01) higher than those of 5.0% polyoxin AS-treated and control fruits (Figure 2c,d). The spray of 0.3% tetramycin AS significantly (*p* < 0.01) inductively enhanced SOD and PPO activities in kiwifruit during storage, the improved effect was significantly (*p* < 0.01) higher than that of 5.0% polyoxin AS. These findings here emphasize that 0.3% tetramycin AS treatment significantly (*p* < 0.01) enhanced total phenolics and total flavonoids contents, as well as SOD and PPO activities of kiwifruit during storage, potentially improving disease resistance.

### 3.5. The Effects of Tetramycin on Growth and Quality of Kiwifruit

The effect of tetramycin and polyoxin on kiwifruit growth is shown in Table 4. 0.3% tetramycin AS application could significantly (*p* < 0.05) enhance transverse diameter of fruits compared to 5.0% polyoxin AS treatment and control. Longitudinal diameter, lateral diameter and fruit shape index of fruits were no significant (*p* < 0.05) differences in three treatments. Moreover, the single fruit volume and weight of kiwifruit treated by 0.3% tetramycin AS were 71.42 cm^3^ and 80.93 g, which was significantly (*p* < 0.05) increased by 2.26 and 4.98%, or 7.34 and 6.22% compared to 5.0% polyoxin AS treatment or control, respectively. Simultaneously, the enhanced effects of fruit growth by 0.3% tetramycin AS was higher than those of 5.0% polyoxin AS. These results indicate that 0.3% tetramycin AS application significantly (*p* < 0.05) enhanced fruit growth and yield formation.

**Figure 3:**
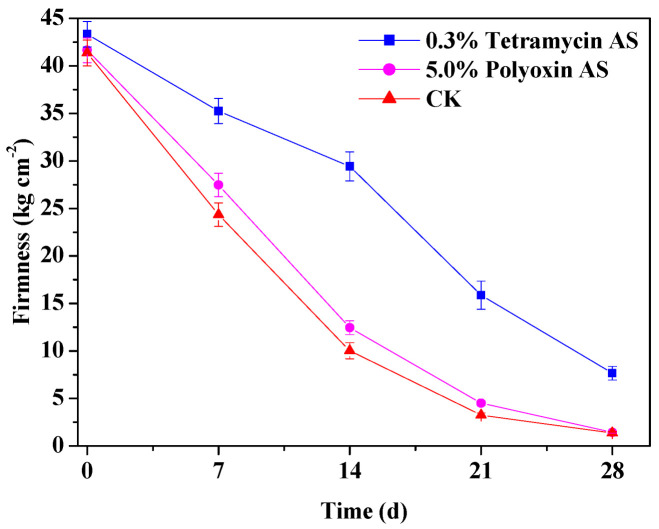
The effects of tetramycin and polyoxin on firmness of kiwifruit during storage. Values indicate the mean of three replicates, error bars indicate SD of three replicates.

Table 5 displays the nutritional parameters of kiwifruits. 0.3% tetramycin AS application significantly (*p* < 0.05) increased vitamin C, total soluble sugar, soluble solid, dry matter and soluble protein of fruits, as well as decreased titratable acidity of fruits compared to 5.0% polyoxin AS treatment and control. While 5.0% polyoxin AS treatment only significant (*p* < 0.05) increased dry matter of fruits compared to control. As shown in Figure 3, firmness decreased more rapidly in 5.0% polyoxin AS treatment and control than in 0.3% tetramycin AS-treated fruit, 0.3% tetramycin AS application effectively maintained higher fruit firmness and delayed its decrease. Meanwhile, the effect of spraying 0.3% tetramycin AS was better than that of 5.0% polyoxin AS. These findings emphasize that 0.3% tetramycin AS application could improve the quality and storability of kiwifruit.

## 4. Discussion

Few reports have discussed the antibacterial activity of tetramycin against the pathogens responsible for plant bacterial diseases. In this study, 0.3% tetramycin AS exhibited significant antibacterial activity against *Psa, P. fulva* and *A. tumefaciens,* three bacterial pathogens in kiwifruit, compared to conventional antibiotics such as 4.0% kasugamycin WP and 3.0% zhongshengmycin WP. The results showed that tetramycin also has a superior antibacterial activity against some other pathogens. Previous studies have reported that the mycelial growth of *Botrytis cinerea*, *Colletotrichum scovillei*, *Pyricularia oryzae*, *Phytophthora capsici* and *Passalora fulva* of fungal pathogen were significantly inhibited by tetramycin [25,26,27,28,29,30]. This study suggests that 0.3% tetramycin AS caused the greatest toxicities of mycelium growth for *B. dothide, Phomopsis* sp., *A. tenuissima*, *A. mellea* and *P. cactorum* of fungal pathogens in kiwifruit, and EC_50_ values of tetramycin were much higher than those of conventional antibiotics polyoxin and kasugamycin. These results extend the antimicrobial spectrum of tetramycin and provide a basis for the control of various kiwifruit diseases.

The superior antimicrobial activity of a pesticide is the premise for its good better field control efficacy on plant diseases. In this study, 0.3% tetramycin AS could significantly enhance the healing rate of disease spots caused by canker, decrease the incidence rates of diseased fruit and flower budd caused by soft rot and blossom blight, as well as decrease the disease index caused by brown spot. Satisfactorily, the application of 0.3% tetramycin AS exhibited good field control efficacies for canker, soft rot, blossom blight and brown spot disease of kiwifruit with 74.45, 83.55, 84.74 and 89.62%, which were significantly (*p* < 0.01) higher than those of conventional antibiotics 3.0% zhongshengmycin WP or 5.0% polyoxin AS. Song et al. shown that tetramycin had effective protective and curative activity against *B. cinerea* [26], and the aforementioned results also confirm that tetramycin is an agricultural antibiotic with both protective and curative functions against plant diseases. Moreover, tetramycin, a mixture polyene antibiotic, has excellently antimicrobial activity and unique mode of action, which are unlikely to lead to resistance development [30,32]. The results show that 0.3% tetramycin AS could effectively control various kiwifruit diseases, which successfully achieves the multi-purpose effects of one pesticide and availably reduces the use of various pesticides in kiwifruit production, as well as better implements the “double reduction of chemical fertilizers and pesticides” action in China. In order to evaluate the potential risk of tetracycline application on the imbalance of microbial population in kiwifruit, it is thus essential to study the microbial composition of kiwifruit leaves, stems and fruits after tetracycline application in the future researches.

Phenolics and flavonoids, as two important types of plant secondary metabolites, play extremely important roles in systemic resistance of plants [16]. In this study, 0.3% tetramycin AS significantly (*p* < 0.01) enhanced total phenolics and total flavonoids contents of kiwifruit during storage. The findings emphasize that 0.3% tetramycin AS application promoted the healthy growth of kiwifruit plants and fruits, as well as it possibly induced systemic resistance to inhibit disease development by plant or fruit. SOD and PPO are important defense enzymes associated with plant disease resistance [16]. Zhong et al. [33] reported that tetramycin could induce plant disease resistance by activating PPO, phenylalanine ammonia lyase (PAL) and perox-idase (POD) activities. The results presented here show that 0.3% tetramycin AS significantly enhanced SOD and PPO activities in kiwifruit during storage and increased the disease resistance of fruit, which is consistent with the above report. Moreover, 0.3% tetramycin AS application could significantly (*p* < 0.05) enhance transverse diameter, volume, weight, vitamin C, total soluble sugar, soluble solid, dry matter and soluble protein of fruits and decrease titratable acidity of fruits, as well as maintain high fruit firmness and delay its decrease. These findings imply that 0.3% tetramycin AS application could reliably decrease the occurrence of various kiwifruit diseases, and then promote its favorable growth, yield increase, quality and storability improvement.

Tetramycin is a novel 26-member tetraene macrolide antibiotic with environmental friendliness and low toxicity. Generally, kiwifruit cannot be eaten directly after harvest because of it has a soft ripening period of more than 20 d at room temperature. In this study, the extremely low 0.3% tetramycin AS application concentration (5000 times dilution liquid), long safety interval period (1 August to 1 October, 60 d) and long soft ripening period (more than 20 d) could effectively eliminate tetramycin residues in kiwifruit. The food safety risks caused by tetramycin are extremely low, and the public can rest assured to eat kiwifruit. Therefore, tetramycin can be used as a preferred alternative to conventional antibiotics in kiwifruit production, and 0.3% tetramycin AS 5000 times dilution liquid is a safe, beneficial and suitable concentration.

## 5. Conclusions

In conclusion, the present study demonstrates that tetramycin has superior antimicrobial activity against eight kiwifruit pathogens, which much higher than that of the conventional antibiotics polyoxin, kasugamycin and zhongshengmycin. 0.3% Tetramycin AS could effectively control the canker, soft rot, blossom blight and brown spot diseases in kiwifruit, and remarkably enhance phenolics and flavonoids contents, as well as SOD and PPO activities of kiwifruit during storage. Moreover, 0.3% tetramycin AS could reliably enhance fruit growth, quality and storability of kiwifruit. This study highlights that the tetramycin is a preferred alternative to conventional antibiotics for controlling kiwifruit diseases.

## Figures and Tables

**Table 1:** Toxicities of different antibiotics against eight pathogens of kiwifruit.

Diseases	Pathogens	Antibiotic Bactericides	Regression Equation	Determination Coefficient (*R*^2^)	EC_50_ (mg kg^−1^)
Canker	*Psa*	0.3% Tetramycin AS	*y* = 4.9514 + 0.5947*x*	0.9839	1.21
		4.0% Kasugamycin WP	*y* = 1.9636 + 1.4521*x*	0.9098	123.31
		3.0% Zhongshengmycin WP	*y* = 3.6935 + 1.0628*x*	0.9923	16.95
Soft rot	*B. dothidea*	0.3% Tetramycin AS	*y* = 6.0759 + 1.2511*x*	0.9963	0.14
		5.0% Polyoxin AS	*y* = 2.0651 + 1.5223*x*	0.9931	84.75
		4.0% Kasugamycin WP	*y* = 3.1542 + 0.6457*x*	0.9652	722.21
	*Phomopsis* sp.	0.3% Tetramycin AS	*y* = 1.1510 + 9.3601*x*	0.9968	0.09
		5.0% Polyoxin AS	*y* = 2.3579 + 1.6551*x*	0.9684	39.48
		4.0% Kasugamycin WP	*y* = 2.6214 + 0.7634*x*	0.9857	1306.36
Blossom blight	*P. fulva*	0.3% Tetramycin AS	*y* = 4.9404 + 0.6414*x*	0.9744	1.24
		4.0% Kasugamycin WP	*y* = 3.6968 + 0.6237*x*	0.9144	122.91
		3.0% Zhongshengmycin WP	*y* = 3.1389 + 0.7711*x*	0.9040	259.24
Brown spot	*A. tenuissima*	0.3% Tetramycin AS	y = 5.7631 + 0.9705*x*	0.9928	0.16
		5.0% Polyoxin AS	*y* = 2.3767 + 2.2548*x*	0.959	14.57
		4.0% Kasugamycin WP	*y* = 4.1000 + 0.2867*x*	0.9432	1362.11
Crown gall	*A. tumefaciens*	0.3% Tetramycin AS	*y* = 7.3724 + 1.0031*x*	0.9962	0.72
		4.0% Kasugamycin WP	*y* = 3.5950 + 0.6408*x*	0.9813	519.24
		3.0% Zhongshengmycin WP	*y* = 4.1821 + 0.2371*x*	0.9759	2811.88
Root rot	*A. mellea*	0.3% Tetramycin AS	*y* = 5.6952 + 0.5530*x*	0.9922	0.06
		5.0% Polyoxin AS	*y* = 4.3471 + 0.5381*x*	0.9866	16.34
		4.0% Kasugamycin WP	*y* = 2.8248 + 0.7971*x*	0.9801	558.41
	*P. cactorum*	0.3% Tetramycin AS	*y* = 5.2013 + 0.2600*x*	0.9942	0.17
		5.0% Polyoxin AS	*y* = 4.5736 + 0.4337*x*	0.9915	9.62
		4.0% Kasugamycin WP	*y* = 0.8490 + 1.4371*x*	0.9920	793.17

*x* and *y* indicate the concentration of antibiotic bactericide and the inhibition rate of bacteria or fungal, respectively.

**Table 2:** The control effects of tetramycin and zhongshengmycin on canker disease of kiwifruit.

Treatments	Healing Rate of Disease Spots (%)	Control Effect (%)
0.3% Tetramycin AS	72.68 ± 3.46 ^aA^	74.45 ± 3.61 ^aA^
3.0% Zhongshengmycin WP	50.23 ± 2.97 ^bB^	45.24 ± 3.32 ^bB^
CK	3.95 ± 0.38 ^cC^	

Values indicate the mean of three replicates ± standard deviation (SD). Different uppercases and lowercases indicate significant differences between different treatments at 1% level (*p* < 0.01) and 5% level (*p* < 0.05), respectively.

**Table 3:** The control effects of tetramycin and polyoxin on soft rot, blossom blight and brown spot diseases of kiwifruit.

Treatments	Soft Rot		Blossom Blight		Brown Spot	
	Incidence Rate of Disease Fruit(%)	Control Effect (%)	Incidence Rate of Disease Flower Bud (%)	Control Effect (%)	Disease Index	Control Effect (%)
0.3% Tetramycin AS	9.00 ± 2.65 ^cC^	83.55 ± 3.47 ^aA^	5.25 ± 0.75 ^cC^	84.74 ± 2.60 ^aA^	3.21 ± 0.99 ^cC^	89.62 ± 2.56 ^aA^
5.0% Polyoxin AS	21.00 ± 1.73 ^bB^	60.83 ± 4.63 ^bB^	22.56 ± 2.28 ^bB^	34.73 ± 5.40 ^bB^	13.67 ± 1.86 ^bB^	55.51 ± 3.31 ^bB^
CK	54.00 ± 6.24 ^aA^		34.56 ± 2.03 ^aA^		30.63 ± 1.96 ^aA^	

Values indicate the mean of three replicates ± standard deviation (SD). Different uppercases and lowercases indicate significant differences between different treatments at 1% level (*p* < 0.01) and 5% level (*p* < 0.05), respectively.

**Table 4:** The effects of tetramycin and polyoxin on the development of kiwifruit.

Treatments	Longitudinal Diameter (mm)	Transverse Diameter (mm)	Lateral Diameter (mm)	Fruit Shape Index	Single Fruit Volume (cm^3^)	Single Fruit Weight (g)
0.3% Tetramycin AS	76.60 ± 1.05 ^a^	53.15 ± 0.51 ^a^	41.91 ± 0.82 ^a^	1.61 ± 0.02 ^a^	71.42 ± 1.06 ^a^	80.93 ± 0.86 ^a^
5.0% Polyoxin AS	76.14 ± 0.74 ^a^	51.57 ± 0.50 ^b^	42.49 ± 0.70 ^a^	1.62 ± 0.03 ^a^	69.84 ± 1.69 ^b^	77.10 ± 0.14 ^b^
CK	75.24 ± 0.17 ^ab^	51.05 ± 0.38 ^b^	41.35 ± 0.09 ^a^	1.63 ± 0.01 ^a^	66.50 ± 0.68 ^c^	76.19 ± 0.80 ^bc^

Values indicate the mean of three replicates ± standard deviation (SD). Different lowercases indicates significant differences between different treatments at 5% level (*p* < 0.05).

**Table 5:** The effects of tetramycin and polyoxin on the nutritional quality of kiwifruit.

Treatments	Vitamin C (g kg^−1^)	Total Soluble Sugar (%)	Soluble Solid (%)	Dry Matter (%)	Soluble Protein (%)	Titratable Acidity (%)
0.3% Tetramycin AS	1.89 ± 0.01 ^a^	12.44 ± 0.11 ^a^	1.89 ± 0.01 ^a^	19.50 ± 0.05 ^a^	1.76 ± 0.05 ^a^	1.01 ± 0.01 ^b^
5.0% Polyoxin AS	1.83 ± 0.01 ^ab^	12.08 ± 0.02 ^b^	1.82 ± 0.01 ^b^	19.25 ± 0.10 ^a^	1.74 ± 0.01 ^a^	1.12 ± 0.06 ^a^
CK	1.81 ± 0.01 ^b^	12.07 ± 0.02 ^b^	1.81 ± 0.01 ^b^	18.39 ± 0.08 ^b^	1.71 ± 0.01 ^ab^	1.13 ± 0.03 ^a^

Values indicate the mean of three replicates ± standard deviation (SD). Different lowercases indicates significant differences between different treatments at 5% level (*p* < 0.05).

## Data Availability

The data used to support the findings of this study are available from the corresponding author upon request.

## Supplementary Materials

The following are available online at https://www.mdpi.com/2079-6382/10/3/289/s1. Figure S1. The plot distribution figure of field experiment of kiwifruit canker. Figure S2. The plot distribution figure of field experiment of soft rot, blossom blight and brown spot diseases in kiwifruit.
