# Supplementary material for: Bioactivity and Control Efficacy of the Novel Antibiotic Tetramycin against Various Kiwifruit Diseases
Source: Antibiotics (Basel). 2021 Mar 10;10(3):289. doi: 10.3390/antibiotics10030289 (PMC7998335; doi:10.3390/antibiotics10030289)
Supplement: Supplementary file 1 [file antibiotics-10-00289-s001.pdf]

<table>
<tr><td rowspan="7">Protection row</td><td colspan="5">Protection row</td><td rowspan="7">Protection row</td></tr>
<tr><td>A1</td><td>Protection row</td><td>B1</td><td>Protection row</td><td>C2</td></tr>
<tr><td colspan="5">Protection row</td></tr>
<tr><td>C3</td><td>Protection row</td><td>A3</td><td>Protection row</td><td>B3</td></tr>
<tr><td colspan="5">Protection row</td></tr>
<tr><td>B2</td><td>Protection row</td><td>C1</td><td>Protection row</td><td>A2</td></tr>
<tr><td colspan="5">Protection row</td></tr>
</table>

Figure S1. The plot distribution figure of field experiment of kiwifruit canker. A: 0.3% tetramycin AS 50 times dilution liquid, B: 3.0% zhongshengmycin WP 50 times dilution liquid and C: clear water (control).

<table>
<tr><td rowspan="7">Protection row</td><td colspan="5">Protection row</td><td rowspan="7">Protection row</td></tr>
<tr><td>B3</td><td>Protection row</td><td>A2</td><td>Protection row</td><td>B2</td></tr>
<tr><td colspan="5">Protection row</td></tr>
<tr><td>C2</td><td>Protection row</td><td>C1</td><td>Protection row</td><td>A1</td></tr>
<tr><td colspan="5">Protection row</td></tr>
<tr><td>A3</td><td>Protection row</td><td>B1</td><td>Protection row</td><td>C3</td></tr>
<tr><td colspan="5">Protection row</td></tr>
</table>

Figure S2. The plot distribution figure of field experiment of soft rot, blossom blight and brown spot diseases in kiwifruit. A: 0.3% tetramycin AS 5000 times dilution liquid, B: 5.0% polyoxin AS 5000 times dilution liquid and C: clear water (control).
